# Antioxidant and Antibacterial Activity of Roseroot (*Rhodiola rosea* L.) Dry Extracts

**DOI:** 10.3390/molecules23071767

**Published:** 2018-07-18

**Authors:** Olga Kosakowska, Katarzyna Bączek, Jarosław L. Przybył, Ewelina Pióro-Jabrucka, Weronika Czupa, Alicja Synowiec, Małgorzata Gniewosz, Rosaria Costa, Luigi Mondello, Zenon Węglarz

**Affiliations:** 1 Laboratory of New Herbal Products, Department of Vegetable and Medicinal Plants, Faculty of Horticulture, Biotechnology and Landscape Architecture, Warsaw University of Life Sciences SGGW, 02-776 Warsaw, Poland; katarzyna_baczek@sggw.pl (K.B.); jaroslaw_przybyl@sggw.pl (J.L.P.); ewelina_pioro_jabrucka@sggw.pl (E.P.-J.); w.czupa@gmail.com (W.C.); zenon_weglarz@sggw.pl (Z.W.); 2 Division of Food Biotechnology and Microbiology, Department of Biotechnology, Microbiology and Food Evaluation, Faculty of Food Sciences, Warsaw University of Life Sciences SGGW, 02-776 Warsaw, Poland; alicja_synowiec@sggw.pl (A.S.); malgorzata_gniewosz@sggw.pl (M.G.); 3 Dipartimento di Scienze Biomediche, Odontoiatriche, e Delle Immagini Morfologiche e Funzionali (BIOMORF), University of Messina, 98122 Messina, Italy; costar@unime.it; 4 Dipartimento di Scienze Chimiche, Biologiche, Farmaceutiche ed Ambientali (ChiBioFarAm), University of Messina, 98122 Messina, Italy; lmondello@unime.it; 5 Chromaleont s.r.l., a Start-Up of the University of Messina, 98122 Messina, Italy

**Keywords:** roseroot, underground organs, dry extracts, salidroside, rosavins, essential oil, geraniol, antioxidant power, antibacterial activity

## Abstract

Roseroot (*Rhodiola rosea* L.) belongs to plants revealing adaptogenic properties, which are attributed to the presence of specific phenolic compounds and are reflected mainly as antioxidant activity. The aim of the present study was to determine the antioxidant and antibacterial activity of various products obtained from *R. rosea* (underground organs as well as their aqueous and ethanolic dry extracts) in relation to the chemical profiles of phenolic and essential oil compounds. The chemical profiles were determined by High-performance Liquid Chromatography with a diode-array detector (HPLC-DAD) and Gas chromatography-mass spectrometry (GC-MS), antioxidant activity by (1,1-Diphenyl-2-picrylhydrazyl) Scavenging Capacity Assay (DPPH), (2,2′-Azino-bis(3-ethylbenzothiazoline-6-sulphonic acid)) Scavenging Capacity Assay (ABTS) and Ferric Reducing Antioxidant Power Assay (FRAP) and antimicrobial properties were expressed as minimum inhibitory concentration (MIC) and minimum bacterial concentration (MBC) values following the broth microdilutions method. The results show that the investigated samples differed in terms of their chemical compositions and biological activities. The extracts were more abundant in phenolic compounds (salidroside, tyrosol, and rosavin derivatives) in comparison to dried underground organs. The content of the determined phenolics in the analyzed extracts was affected by the solvent used for extraction. The ethanolic extract was characterized by the highest content of these substances in comparison to the aqueous one and the dried raw material, especially with regard to rosavin (969.71 mg/100 g). In parallel, this extract showed the strongest antioxidant and antibacterial activity. However, dried *R. rosea* underground organs also revealed strong antibacterial effects against, for example, *Staphylococcus* strains.

## 1. Introduction

Plants belonging to the *Rhodiola* genus (*Crassulaceae* family) are distributed mainly throughout the arctic zone of the Northern hemisphere. They also occur in the mountainous regions of Asia, Europe, and North America, and can be classified as arctic-alpine oreophytes. The genus consists of nearly 200 species, of which approximately 20 are used as traditional medicine in Asia, for example, *Rhodiola kirilovii*, *R. crenulata*, *R. sacra*, *R. alterna*, *R. quadrifia,* and *R. rosea* [1,2]. *R. rosea*, the so-called “golden root” or “roseroot” is the most widely known and investigated species. Its underground organs (rhizomes and roots) have been used for ages in the Far East as a natural remedy to eliminate fatigue, improve memory, and enhance physical and mental performance as well as to prevent high-altitude sickness. It also has been used in the treatment of heart diseases, depression, and anxiety, and is especially recommended for hard-working people, convalescents, and elderly people [1,2,3]. This raw material is mainly rich in phenylethanoids, phenylpropanoid glycosides, and other phenolic compounds, including phenolic acids, flavonoids, and tannins. Phenylethanoids are represented here by tyrosol derivatives, *p*-tyrosol and salidroside, while among phenylpropanoid glycosides, there are *trans*-cinnamic acid derivatives, such as rosarin, rosavin, rosin, and *trans*-cinnamic alcohol. *Trans*-cinnamic acid derivatives, classified together under the name “rosavins” are specific for *R. rosea* and are not detected in other *Rhodiola* species [3,4,5]. In the group of phenolic acids, chlorogenic, hydroxycinnamic, and gallic acids have been found, while within flavonoids, there are *Rhodiola*-specific compounds, such as rhodionidin, rhodiolgin, rhodalidin, rhodionin, and rhodalin as well as kaempferol and tricin derivatives. The presence of proanthocyanidins (prodelphinidin-gallate esters), flavonolignans (rhodiolin), and cyanogenic glycosides (rhodiocyanoside A) has been reported as well [3,4,5,6,7]. Roseroot also contains 0.05% essential oils, which are responsible for the specific rose-like flavor of this raw material. Here, the dominant compounds are *n*-decanol, geraniol, rosiridol, rosiridin, 1.4-menthadien-7-ol, geranyl acetate, benzyl alcohol, and phenylethyl alcohol [8,9,10,11].

Due to having such a wide range of biologically active compounds, roseroot has various pharmacological activities, i.e., antioxidant, anti-inflammatory, anti-fatigue, anti-depressive, anxiolytic, anti-tumor, anti-microbial, neuro- and cardioprotective, and normalizing endocrine and immune activity. Given such properties, *R. rosea* has been classified as a plant adaptogen [1,2,3,12]. Taking into account the definition of an adaptogen (a substance that enables an increase in the human body’s resistance to various chemical, biological, and physical stressors), roseroot is regarded as one of the most active [3,4,13]. In this instance, its antioxidant potential seems to be directly associated with its adaptogenic activity. Reactive oxygen species (ROS), so-called free radicals, are highly chemically active forms of oxygen that have significant impacts on cells. At low levels, they modulate gene expression and cell proliferation and can also induce transcription and apoptosis. Moreover, they participate in inflammatory and angiogenic processes. However, these substances can be overproduced as a result of oxidative stress. Thus, in high concentrations, free radicals can damage all major cell components: cell membranes, lipids, proteins, and nucleic acids. This could be a reason for the development of various diseases, for example, cancer, arteriosclerosis, hypertension, obesity, and Parkinson’s and Alzheimer’s diseases, with the development of civilization [2,14]. Recently, substances that delay or prevent oxidation (so-called antioxidants) have been under intense study. The number of antioxidant compounds synthesized by plants as secondary products, mainly phenolics, serving in plant defense mechanisms to counteract ROS to survive, is currently estimated to be between 4000 and 6000 [15]. In the case of *R. rosea*, numerous works have indicated that its raw materials and various extracts have high antioxidative potential [16,17,18,19,20,21].

*R. rosea* roots and rhizomes can be used both in the form of crude drug (dried, powdered underground organs) and/or water/alcoholic extracts. According to the European Medicines Agency [4], the following three types of extracts are applied in official medicinal use: dry extract (DER 2.5–5:1) obtained by double step extraction (70% ethanol, water), dry extract (DER 1.5–5:1) obtained with 60% ethanol, and liquid extraction (DER 1:1) obtained with 40% ethanol. A single dose and treatment duration are specified for the abovementioned extracts. Recently, roseroot has become very popular, not only in pharmaceutical preparations, but also in food additives, diet supplements, and drinks offered worldwide. Thus, various herbal products made from *R. rosea* underground organs are available and traded in the markets [2,11]. However, the quality of such products, including chemical composition and biological activity (i.e., antioxidant potential), is usually unknown.

The aim of the present study was to determine the antioxidant and antibacterial activity of underground organs of *R. rosea* as well as their aqueous and ethanolic dry extracts in relation to the chemical profiles of phenolic and essential oil compounds.

## 2. Results and Discussion

### 2.1. Composition of Phenolic Compounds

The samples investigated were evaluated in terms of the content of the following substances: tyrosol derivatives (salidroside and its aglycon tyrosol) as well as rosavins (*trans*-cinnamic alcohol derivatives, including rosavin, rosarin, rosin, and *trans*-cinnamic alcohol) (Table 1). Because these compounds are regarded to be responsible for the pharmacological activity of *R. rosea*, they were used to standardize the raw materials [5,22]. The content of tyrosol derivatives and rosavins in *R. rosea* underground organs depends on various factors, i.e., harvest time, age of the plant, its origin, method of raw material stabilization, and extraction method [23]. The samples examined in this work differed as to the content of listed compounds. In general, dry extracts, being more concentrated, were more abundant in phenolic compounds (except *trans*-cinnamic alcohol) in comparison to powdered raw material. Here, the amounts of certain compounds were clearly affected by the solvent used for extraction. Salidroside and rosarin dominated in powdered raw material (146.30 and 141.03 mg/100 g, respectively); in aqueous extract, salidroside was present in the highest amount (249.87 mg/100 g), while, in the ethanolic one, rosavin dominated (969.71 mg/100 g). The ethanolic extract was characterized by significantly greater contents of the compounds studied than the aqueous extract, especially in regard to rosavin (969.71 mg/100 g and 129.15 mg/100 g^−1^, respectively). Previous data concerning the recommendations of solvent and extraction methods seem to be contradictory. According to Altantsetseg et al. [24], water appears to be the best extraction medium for salidroside and tyrosol. However, Węglarz et al. [23] claimed that the highest content of salidroside can be obtained when 70% ethanol is used. Results by Kucinskaite [6] show that, for salidroside, it is better to use 40% ethanol than 70%. Listed authors recommended 70–75% ethanol to isolate rosavins.

### 2.2. Essential Oil Content and Composition

Because the analyzed extracts contained only traces of the volatile compounds, the total content and composition of essential oil were determined only for the raw material. It was measured to be at a level of 0.07 g/100 g, which corresponds with results obtained by other authors [8,10]. In all, 27 compounds were identified in the study, comprising 95.09% of the total identified fractions. The following were the dominant substances: tetrahydronootkatone (18.31%), geraniol (16.46%), *trans*-pinocarveol (15.71%), octanol (11.43%), and myrtenol (11.29%). *Trans*-carveol, myrtanol, and perilla-alcohol were also present in considerable amounts (Table 2). In general, most of the abovementioned compounds have been reported earlier by other authors [8,9,10,11]. According to Hethelyi et al. [10], geraniol, myrtenol, and linalool are responsible for the pleasant rose-like aroma of *R. rosea*’s underground organs. In his study, Rohloff [8] listed geranyl formate, geranyl acetate, benzyl alcohol, and phenylethyl alcohol as well. Results obtained by Shatar et al. [9] indicated the domination of geraniol (32.3%), myrtenol (15.7%), and octanol (13.7%) in the essential oil from *R. rosea*. However, Rohloff [8] showed that the amount of *n*-decanol (30.38%) was the highest, while, in the studies by Hethelyi et al. [10], it was myrtenol (36.9%). The results of Evstatieva et al.’s [11] study showed significant differences in the composition of *R. rosea* essential oil originating from plants growing in various locations (e.g., 56.97% of geraniol in the sample from China; 56.22% of phenylethyl alcohol—from India). Considering such variability, it is suspected that *R. rosea* creates different essential oil chemotypes. The sample examined in the present work represents a mixed (tetrahydronootkatone/geraniol/*trans*-pinocarveol) chemotype.

### 2.3. Antioxidant Activity

The DPPH and ABTS scavenging assays and the ferric reducing antioxidant power (FRAP) assay are based on single electron transfer (SET) reactions. In the DPPH and ABTS assays, this reaction results in a decrease in the absorbance of free radical species, visible as the change of color from purple (in the case of DPPH) and green/blue (in the case of ABTS) to yellow, whereas the FRAP assay relies on the ability of antioxidants to reduce Fe^3+^ to Fe^2+^ in the presence of tripyridyltriazine (TPTZ), which results in the formation of an intense blue Fe^2+^–TPTZ complex [27,28].

The results obtained in this work showed that the *R. rosea* ethanolic extract possesses the strongest antioxidant activity (DPPH—85.90%; ABTS—89.60%; FRAP—1198.20 Fe^2+^ µmol/g) compared to the aqueous extract or powdered raw material (Table 3). Such a result can be attributed to the fact that this extract was distinguished by the highest level of phenolic compounds, particularly salidroside, which is regarded as a strong antioxidant. According to Quian et al. [29], salidroside-reduced hydrogen peroxide induces intracellular ROS production in human erythrocytes. Results by Mao et al. [30] showed that salidroside can prevent morphological changes in cultured human fetal lung fibroblasts after sublethal doses of hydrogen peroxide. However, as per recent studies, the aglycon of salidroside (tyrosol) does not possess antioxidant activity [31].

Given the results obtained, evidently the dried raw material (underground organs of *R. rosea*) was distinguished by the lowest antioxidant activity (Table 3). This fact can be associated with the presence of volatile fractions within this sample. According to some authors, essential oils can reveal antioxidant activity, especially those rich in phenolics (e.g., thymol and/or carvacrol) [32,33]. However, such activity of essential oils is rather low, particularly when compared to polar extracts [15,34,35]. Thus, the results obtained in this study suggest that the presence of essential oil in *R. rosea* underground organs is not related to the antioxidant activity of this raw material. 

### 2.4. Antibacterial Activity

Apart from their antioxidant potential, the *R. rosea* underground organs revealed antibacterial activity. However, the data from the literature with respect to this problem is rather scarce. In this study, the MIC and MBC values of plant preparations, that is, powdered raw material, aqueous and ethanolic extracts obtained from *R. rosea* as an assessment of their activity against selected pathogenic bacteria have been determined. The serial microdilution method has been used in research and is useful for screening many combinations of various bacteria and plant preparations. It is also economical in terms of time and means [36].

The tested *R. rosea* preparations had bacteriostatic and bactericidal activity against all test bacteria in the range from 1 to 64 mg/mL (except powdered raw material and aqueous extract) (Table 4). However, differences in the effects of individual preparations on bacteria were observed. For the tested plant preparations, the most susceptible bacteria were found to be *Staphylococcus epidermidis* (MIC 1–2 mg/mL, MBC 1–8 mg/mL) and *Klebsiella pneumoniae* (MIC/MBC 1–4 mg/mL).

Powdered raw material showed a weaker antibacterial effect than the two other tested preparations. The MICs of this preparation in relation to the majority of test strains ranged from 16 to 32 mg/mL, and MBCs were in the concentration range of 16‒64 mg/mL. This preparation inhibited only the *Staphylococcus* strains (MIC 1‒8 mg/mL, MBC 4‒8 mg/mL) and *K. pneumoniae*, *Yersinia enterocolitica*, *Shigella sonnei*, and *Proteus mirabilis* (MIC/MBC 2–8 mg/mL) more strongly. Such results may be associated with the presence of essential oil in this sample. One of its dominant compounds, geraniol, is regarded to be strongly active against *Staphylococcus aureus* [37]. The powdered raw material did not have bactericidal activity on *Listeria monocytogenes*, *Enterobacter aerogenes*, or *Escherichia coli* ATCC 25922 in the concentration range tested (MBC > 64 mg/mL).

The aqueous and ethanolic extracts of *R. rosea* showed stronger bacteriostatic effects than the powdered raw material for most strains of Gram-positive and Gram-negative bacteria. The inhibition by the aqueous extract of the growth of two strains of *Bacillus cereus*, *Bacillus subtilis*, *Salmonella* ser. Enteritidis, *Salmonella* ser. Typhimurium, and *E. coli* was two-fold stronger, and inhibition of *L. monocytogenes* was four-fold stronger compared to the powdered raw material. However, the bactericidal effect of this extract was weaker or the same as the powdered raw material with respect to most test strains, with the exception of *B. cereus* ATCC 11778, *P. mirabilis*, *Pseudomonas aeruginosa*, and *E. coli* ATCC 25922. The aqueous extract also did not indicate bactericidal effect on the two clinical strains of *S. aureus*, and *S.* ser. Typhimurium (MBC > 64 mg/mL). The ethanolic extract from *R. rosea* particularly inhibited the growth of all Gram-positive bacterial strains (MIC 1–4 mg/mL) from other preparations, with the *L. monocytogenes* strain being the most sensitive (MIC 4 mg/mL, MBC 8 mg/mL). The ethanolic extract also revealed lower MBC values against most Gram-positive strains than the powdered raw material and aqueous extract. Among the Gram-negative bacteria, the ethanolic extract more strongly inhibited the growth of *S. sonnei* and *Ps. aeruginosa* (MIC 4 and 8 mg/mL, respectively) than the powdered raw material and aqueous extract. In contrast, the bactericidal activity against the majority of Gram-negative bacteria strains was the same as the aqueous extract.

On the basis of these results, it can be concluded that strains of Gram-positive bacteria were more sensitive to the preparations of *R. rosea* than Gram-negative bacteria. The reference strains showed greater sensitivity to the test preparations than the clinical strains belonging to the same species (*S. aureus*, *Salmonella enterica*, and *E. coli*). The MBC values of the preparations were, in most cases, greater than the MIC values. The antibacterial activity of *R. rosea* preparations has been the subject of very few studies [38,39,40]. Ming et al. [39] showed that the methanol extract of the underground part of *R. rosea* has inhibitory activity against *Staphylococcus aureus*. The effect of feeding mice with *R. rosea* extracts was studied on *Pseudomonas aeruginosa* infection by Bany et al. [41]. The infection intensity was significantly lower in mice after treatment for 7 days with a daily dose of 0.4 mg of aqueous extract than in the control group.

Table 5 shows the activity of three preparations obtained from *R. rosea* (A). The test preparations were characterized by a diversified spectrum of activity at each concentration tested. Due to the spectrum of activity on bacteria, they can be arranged in the following way: powdered raw material < aqueous extract < ethanol extract. Seventy-four percent of the test strains were inhibited by this extract at a concentration of 8 mg/mL, while powdered raw material at the same concentration inhibited 37% of strains, an aqueous extract of 47%.

## 3. Materials and Methods

### 3.1. Plant Samples

The raw plant material (roots and rhizomes of *R. rosea*) was obtained from the collection of medicinal and aromatic plants belonging to the Department of Vegetable and Medicinal Plants, Warsaw University of Life Sciences, WULS-SGGW, Poland. Seed material originated from wild-growing roseroot populations located in Mongolia. Voucher specimens were deposited at the herbarium of the Department of Vegetable and Medicinal Plants and seeds were deposited in the National Centre for Plant Genetic Recourses (Polish Gene Bank, accession no. 503981). Raw materials were collected in the year 2015 from 3-year-old plants before vegetation (early spring) and were dried at 35 °C and stored in darkness. Three samples were finally subjected to chemical and biological evaluation: *R. rosea* roots and rhizomes (dried, powdered raw material), dry aqueous extract, and dry ethanolic extract.

### 3.2. Preparation of Extracts

The extraction of raw materials was performed by a commercial company Greenvit—Botanical Extracts Manufacturer, Poland. The extracts were prepared in a glass extractor (Megan S.C.) at 50 °C using water or ethanol 60% (*v*/*v*) and a plant-to-solvent ratio of 1:10 (*w*/*v*). The extraction time was 6 h. Afterward, the extracts were filtered through a filter paper using a Buchner funnel connected to a vacuum pump (Vacuubrand 4C) at 500 mmHg. The extracts were concentrated in a rotary evaporator (Heidolph Hei-Vap Value Digital) at a vacuum pressure of 50 mBar and a temperature of 50 °C. Drying was conducted in a vacuum dryer (Binder, VD 53) at a temperature of 60 °C. No drying carriers were used.

### 3.3. Preparation of Essential Oil

The essential oil was isolated from underground organs of *R. rosea* in accordance with *Polish Pharmacopeia* 8th edition, with modifications [42]. Air-dried raw material (100 g) was subjected to hydrodistillation for 3 h using a Clevenger-type apparatus. The amount of essential oil was measured on the apparatus scale. The obtained essential oils were stored in dark vials, at 4 °C.

### 3.4. Chemical Analysis

The chemical compositions of phenolic compounds were determined by HPLC (high pressure liquid chromatography) (Section 3.4.1), while the composition of essential oil was determined by GC-MS (gas chromatography-mass spectrometry) and GC-FID (gas chromatography-flame ionization detector) (Section 3.4.2). All measurements were performed in triplicate.

#### 3.4.1. Analysis of Phenolic Compounds by HPLC

A total of 1.0 g of air-dried, powdered, and homogenized *R. rosea* roots and rhizomes were extracted with 100 mL of methanol using Extraction System B-811 (Büchi Labortechnik AG, Flawil, Switzerland). Soxhlet hot extraction with 25 extraction cycles, flushing, and drying was used. After evaporation of the solvent, the residue was dissolved in 10 mL of methanol. Then, 1.0 g of dry ethanolic and aqueous extracts was dissolved in 10 mL of methanol. All obtained extracts were filtered with a Supelco (Sigma-Aldrich^®^, St. Louis, MO, USA) Iso-Disc™ Syringe Tip Filter Unit and a PTFE membrane with a 25 mm diameter and 0.22 μm pore size.

The quantitation was performed using a Shimadzu chromatograph, equipped with an auto-sampler (SIL-20A (Shimadzu, Kyoto, Japan ly)), photodiode array detector (SPD-M10A VP PDA (Shimadzu, Kyoto, Japan)), and CLASS VP^™^ 7.3 chromatography software (Shimadzu, Kyoto, Japan). Separations were achieved using a C18 reversed-phase, 2.6 μm, 100 mm × 4.60 mm Kinetex^™^ column (ChromaDex^®^, Irvine, CA, USA) with a porous outer layer on solid silica core particles (Phenomenex^®^, Torrance, CA, USA). The binary gradient of deionized water was adjusted to pH 3 with phosphoric acid (Sigma-Aldrich, Poznań, Poland) as phase A and MeCN (Sigma-Aldrich, Poznań, Poland) was used as phase B as follows: 0.01 min at 12% B; 5.50 min at 20% B; 5.51 min at 50% B; 6.50 min at 50% B; 6.51 min at 12% B, and at 11 min, the process stopped. The flow rate was 1.2 mL min^−1^, the oven temperature was 45 °C, and the injection volume was 1 μL.

Commercially available standards (ChromaDex^®^, Irvine, CA, USA) were separately dissolved in methanol (Sigma-Aldrich, Poznań, Poland) in 10 mL volumetric flask according to the ChromaDex’s Tech Tip 0003: Reference Standard Recovery and Dilution and used as standard stock solutions [42]. Working standard solutions were prepared by dilution of 100 µL and 2000 µL stock solutions of each compound with methanol in 10 mL volumetric flasks. The working solutions (1 µL) and stock solutions (1, 2, 5, and 10 μL) were injected on a column in six replicates (*n* = 6) to generate a six-point calibration curve. A peak table and UV-spectra library (190–500 nm) of individual compounds were created. Quantitation was done with analytical wavelength appropriate for each compound: 222 nm for salidroside and tyrosol, and 249 nm for rosarin, rosavin, rosin, and *trans*-cinnamic alcohol. Standard curve parameters and validation parameters were calculated with Microsoft Excel (Table 6). The signal-to-noise (S/N) ratio approach was used to determine the limit of detection (LOD; S/N of 3:1) and limit of quantitation (LOQ; S/N of 10:1). The content of the determined compounds was calculated in mg per 100 g of dry matter.

#### 3.4.2. Analysis of Essential Oils by GC-MS and GC-FID

Qualitative GC-MS analyses were performed on a Shimadzu GCMS-QP2010 gas chromatograph-mass spectrometer (Shimadzu, Milan, Italy) equipped with a Shimadzu autoinjector AOC-20is. Data were collected with *GCMS Labsolution* software (Shimadzu, Milan, Italy). The operational conditions were as follows: the temperature program went from 50 °C (2 min) to 250 °C (10 min) at 3 °C/min. The columns were SLB-5ms (30 m × 0.25 mm i.d. × 0.25 µm film thickness) and Supelcowax-10 (30 m × 0.25 mm i.d. × 0.25 µm film thickness), both supplied by Supelco (Merck, Darmstadt, Germany). The GCMS-QP2010 was equipped with a split-splitless injector (250 °C). An injection volume of 0.4 µL was used in split mode (split ratio 20:1). The inlet pressure was 37.7 kPa. The carrier gas was He delivered at a constant linear velocity (*ū*) of 30 cm × s^−1^. The interface temperature was 250 °C. MS ionization mode was used for electron ionization. The detector voltage was 0.95 kV. The acquisition mass range was 40–350 u. The scan speed was 1666 amu × s^−1^. The acquisition mode was full scan with a scan interval of 0.20 s. The identification of essential oil compounds was based on a comparison with mass spectra from the Mass Spectral Database-FFSNC 2, NIST 11, Wiley 9-and by a comparison with the retention indices (RI) of relative to retention times of a series of *n*-hydrocarbons (C7-C30) with those reported in the literature [25,26].

For quantitative GC-FID analyses, a Shimadzu GC-2010 gas chromatograph (Shimadzu, Milan, Italy) equipped with a Shimadzu autoinjector AOC-20is (Shimadzu, Milan, Italy) was used. The injection parameters and temperature program were the same as reported above for the GC-MS analysis. The column was a Supelcowax-10 (30 m × 0.25 mm i.d. × 0.25 µm film thickness). Inlet pressure 100 kPa. The carrier gas was He delivered at a constant linear velocity (*ū*) of 30 cm/s. The FID (275 °C) gases were H_2_ (flow: 50.0 mL min^−1^); air (flow: 400.0 mL min^−1^); He (as made up, flow: 50.0 mL × min^−1^). The percentage compositions of the essential oils were computed by the normalization method from the GC peak areas.

### 3.5. Antioxidant Activity

#### 3.5.1. DPPH (1,1-Diphenyl-2-picrylhydrazyl) Scavenging Capacity Assay

Measurement of the DPPH radical scavenging activity was performed in accordance with Yen and Chen [43], with modifications concerning the time of reaction, in accordance with Szlachta and Małecka [44]. A quantity of 0.25 g of sample (dry ethanolic extract, dry aqueous extract, and powdered raw material) was dissolved in 5 mL of methanol. Then, 3 mL of methanol and 1 mL of DPPH methanolic solution (0.12 mg/mL) were added to 1 mL of the examined extract. Absorbance was measured on the UV/Vis Shimadzu 1700 PharmaSpec spectrophotometer (Shimadzu, Kyoto, Japan) after 10 min at 517 nm. The blind test extract was replaced by 1 mL of methanol. The antioxidant activity of extracts was calculated as *I* = [(*A*_B_ − *A*_A_)/*A*_B_] × 100, where *I* is DPPH inhibition (%); *A*_B_ is the absorbance of a blank sample (*t* = 0 min); and *A*_A_ is the absorption of the extract solution (*t* = 10 min). Trolox standard in concentrations of 0.3–4.9 μg/mL was used to estimate a standard curve. Results are expressed in μmol Trolox equivalents per gram of extract.

#### 3.5.2. ABTS (2,2′-Azino-bis(3-ethylbenzothiazoline-6-sulphonic acid)) Scavenging Capacity Assay

ABTS (ABTS radical caption) was measured according to the method of Re et al. [45] and Arts et al. [46]. A quantity of 0.25 g of sample (dry ethanolic extract, dry aqueous extract, and powdered raw material) was dissolved in 5 mL of methanol. The stock solution was prepared by stirring 7 mM of ABTS and 2.45 mM (final concentration) of potassium persulfate in water and incubating at room temperature in the dark for 16 h before use. The concentrated ABTS was diluted with phosphate buffered saline (PBS) to a final absorbance of 0.72 (±0.2) at 734 nm. Then, 1 mL ABTS solution was added to 100 μL of examined extracts (2-, 5-, 10-, 50- and 100-fold diluted solutions). The absorbance of ABTS was measured on the UV/Vis Shimadzu 1700 PharmaSpec spectrophotometer after 6 min incubation in dark, at 737 nm. The ability of the test sample to scavenge ABTS was compared to the Trolox standard. The Trolox solutions were prepared in PBS, such that the final concentrations of the standards were 0.0, 1.25, 2.5, 5.0, 7.5, and 10.0 mg/100 mL. The percentage inhibition of ABTS of the test samples was calculated according to the following formula: % inhibition = [(*A*_B_ − *A*_A_)/*A*_B_] × 100, where *A*_B_ is the absorbance of a blank sample, and *A*_A_ is the absorbance of the test sample. The results are expressed in μmol Trolox equivalents per gram of extract.

#### 3.5.3. Ferric Reducing Antioxidant Power Assay (FRAP)

A quantity of 0.25 g of sample (dry ethanolic extract, dry aqueous extract, and powdered raw material) was dissolved in 5 mL of methanol. The working reagent was prepared by mixing acetate buffer (300 mM, pH 3.6), a solution of 10 mM TPTZ (2,4,6-tris(2-pyridyl)-*s*-triazine) in 40 mM HCl, and 20 mM FeCl_3_·6H_2_O at a (*v*/*v*/*v*) ratio of 10:1:1. A total of 100 μL of each properly diluted extract solution was prepared in tubes with 3 mL of working reagent and shaken for 30 s. After 2 h of incubation, the absorbance was read at 593 nm. A series of Trolox and Fe_2_SO_4_ solutions in the concentration ranges of 0–479 μg/mL and 0–1000 μg/mL, respectively, was used to prepare the calibration curve. The results were expressed as Trolox equivalents of antioxidant capacity in μmol Trolox per gram of extract, and Fe^2+^ antioxidant capacity (Fe^2+^ μmol/g of extract) [47,48].

### 3.6. Anti-Microbial Activity

#### 3.6.1. Test Microorganisms and Preparation of Inoculum

*R. rosea* could be employed against bacteria that contaminate food. Reference strains originated from the American Type Culture Collection (ATCC, Manassas, VA, USA), and clinical strains originated from the National Institute of Public Health-National Institute of Hygiene (NIPH-NIH, Warsaw, Poland), and the strain isolated from food originated from the Division of Milk Biotechnology (WULS-SGGW, Warsaw, Poland). The study used eight strains of Gram-positive bacteria (*Bacillus cereus* ATCC 11778, *B. cereus* WULS-SGGW 15, *B. cereus* WULS-SGGW X-13, *Bacillus subtilis* ATCC 6633, *Staphylococcus aureus* ATCC 25923, *Staphylococcus epidermidis* ATCC 12228, *Listeria monocytogenes* NIPH-NIH 17/11, *S. aureus* NIPH-NIH A-529) and 11 strains of Gram-negative bacteria (*Enterobacter aerogenes* ATCC 13048, *Escherichia coli* ATCC 25922, *Klebsiella pneumoniae* ATCC13883, *Proteus mirabilis* ATCC 35659, *Salmonella enterica* subsp. *enterica* serovar Enteritidis ATCC 13076, *Pseudomonas aeruginosa* ATCC 27853, *E. coli* O26 NIPH-NIH 152/11, *S. enterica* subsp. *enterica* serovar Enteritidis NIPH-NIH322/11, *Salmonella enterica* subsp. *enterica* serovar Typhimurium NIPH-NIH300/11, *Yersinia enterocolitica* O3 NIPH-NIH 383/11, *Shigella sonnei* NIPH-NIH s). The bacterial strains were cultured on Mueller–Hinton Agar (MHA, Merck, Darmstadt, Germany) and incubated at 37 °C for 18 h. Bacterial inocula were prepared in sterile 0.85% NaCl (*w*/*v*) solution to reach a population of approximately 1 × 10^8^ CFU/mL.

#### 3.6.2. The Minimum Inhibitory Concentration (MIC) and Minimum Bactericidal Concentration (MBC)

The MIC and MBC of plant preparations were determined using the serial microdilution method (CLSI, 2009). Two series of dilutions were prepared in Müller–Hinton Broth (Merck, Darmstadt, Germany) of plant samples in the concentration range of 0.5–64 mg/mL using polystyrene 96-well plates, each of a volume of 200 µL (Primo, ScholaGene, Krakow, Poland). Test bacterial inoculum was added to each well of the plate, so that the final number in 1 mL was 5 × 10^5^. The wells containing inoculated and non-inoculated broth were prepared as growth and purity controls simultaneously. The plates with bacteria were incubated at 37 °C for 24 h. The MIC value was defined as the lowest concentration of plant preparation in which no visual growth of bacteria compared with the plant sample-free control was found, and is expressed in mg/mL. The MIC examination of plant preparations was repeated three times. The MBC examination involved the transfer of 100 μL of bacterial culture from each well where no growth was observed, to the plates with Müller–Hinton Agar (Merck, Darmstadt, Germany). The plates were incubated at 37 °C for 24 h. The growth of colonies on the plates was verified after this time. The MBC was defined as the lowest concentration of plant preparation which resulted in complete inhibition of bacterial growth, and is expressed in mg/mL.

The percentage value of antimicrobial activity of plant preparation was determined based on MIC values (A) [49]:

A (%) = (100 × number of strains inhibited by the examined preparation)/(total number of tested strains)

The percentage of activity proves the complete antimicrobial potency of a particular plant preparation, i.e., it determines the number of bacterial strains susceptible to one specific plant preparation.

### 3.7. Statistical Analyses

Data were subjected to statistical analyses using Statgraphics^®^ plus software (Statgraphics Technologies Inc., The Plains, Virginia). The mean values were compared using the one-way analysis of variance (ANOVA) and are expressed as means with standard deviations (SD). The differences between individual means are signed as various letters in table rows and were considered to be significant at *p* < 0.05.

## 4. Conclusions

*R. rosea* has become very popular in recent years. Although the European Medicines Agency recommends only particular *R. rosea* extracts to be used for medicinal purposes, there are many other preparations available and being traded in markets in the form of food additives or diet supplements. Thus, the purpose of the presented investigation was to characterize three various *R. rosea* products that could be potentially used in the herbal or food industries. Examined samples (powdered underground organs as well as their aqueous and ethanolic dry extracts) differed considerably in terms of chemical composition and biological activity. The ethanolic extract was characterized by a significantly higher content of determined phenolic compounds (salidroside, tyrosol, and rosavin derivatives) than the aqueous one and the raw material, especially with regard to rosavin. This extract revealed the strongest antioxidant and antibacterial activity as well. However, the raw material also revealed a strong antibacterial effect against, for example, *Staphylococcus* strains. Based on data from the literature, it is suspected that such results may be related to the presence of essential oil in this product.

## Figures and Tables

**Table 1:** The chemical compositions of phenolic compounds (mg/100 g).

Compound	Raw Material	Aqueous Dry Extract	Ethanolic Dry Extract
Tyrosol derivatives			
Salidroside	146.30 ± 25.94 ^c^	249.87 ± 1.65 ^b^	441.29 ± 4.67 ^a^
Tyrosol	36.20 ± 0.25 ^b^	136.08 ± 0.37 ^a^	147.08 ± 0.48 ^a^
Total	182.50	385.95	588.37
*Trans*-cinnamic alcohol derivatives			
Rosarin	141.03 ± 3.60 ^c^	181.00 ± 2.27 ^b^	285.48 ± 3.54 ^a^
Rosavin	48.01 ± 1.68 ^c^	129.15 ± 0.12 ^bc^	969.71 ± 3.76 ^a^
Rosin	102.24 ± 2.64 ^b^	127.77 ± 0.98 ^b^	169.40 ± 1.66 ^a^
*Trans*-cinnamic alcohol	17.29 ± 0.38 ^a^	8.90 ± 0.09 ^b^	12.07 ± 0.40 ^ab^
Total	308.57	446.82	1436.66

Means marked in rows with different letters differ at *p* < 0.05.

**Table 2:** The total content (g/100 g) and gas chromatographic composition of essential oil (% area).

No.	Compound	RI ^a^	RI ^b^	Essential Oil
1	hexanal	1058	801	1.33
2	2-pentyl furan	1209	990	0.49
3	pentyl alcohol	1224	763	0.03
4	hexanol	1332	866	0.82
5	*trans*-ocimene	1385	1092	0.08
6	β-thujone	1425	1119	0.06
7	1-octen-3-ol	1434	978	0.09
8	heptanol	1440	969	0.17
9	1-nonanol	1500	1036	0.32
10	benzaldehyde	1511	-	0.23
11	linalool	1537	1100	0.42
12	octanol	1548	1071	11.43
13	pinocarvone	1558	1163	0.10
14	myrtenal	1621	-	0.11
15	*trans*-pinocarveol	1649	1142	15.71
16	decyl alcohol	1767	1274	0.21
17	cuminaldehyde	1783	1244	0.18
18	myrtenol	1797	1198	11.29
19	*trans*-carveol	1842	1221	4.51
20	geraniol	1856	1252	16.46
21	*cis*-myrtanol	1883	1186	5.26
22	perilla-alcohol	2025	1301	2.27
23	(*Z*)-cinnamaldehyde	2058	-	1.38
24	mentha-1.4-dien-7-ol	2078	1194	1.88
25	cumin alcohol	2127	1293	1.32
26	tetrahydronootkatone	2266	1734	18.31
27	(*E*)-cinnamyl alcohol	2322	1311	0.63
	Total identified fraction			95.09
	Essential oil total content			0.07

^a^ Kováts retention indices calculated on polar column Supelcowax 10. ^b^ Kováts retention indices calculated on unpolar column SLB 5 ms, according to [25,26].

**Table 3:** The results of antioxidant activity analyses in vitro (DPPH, ABTS, FRAP).

Method		Raw Material	Aqueous Dry Extract	Ethanolic Dry Extract
DPPH				
	[% RSC]	62.70 ± 1.1 ^b^	79.30 ± 2.0 ^ab^	85.90 ± 3.1 ^a^
	[µmol Trolox/g]	182.70 ± 2.2	229.00 ± 1.1	240.50 ± 0.7
ABTS				
	[% RSC]	74.00 ± 2.4 ^b^	88.70 ± 1.1 ^a^	89.60 ± 0.5 ^a^
	[µmol Trolox/g]	36.80 ± 2.5	43.60 ± 1.5	45.80 ± 1.1
FRAP				
	[Fe^2+^ µmol/g]	399.40 ± 2.1 ^c^	542.60 ± 1.0 ^b^	1198.20 ± 0.7 ^a^
	[µmol Trolox/g]	204.90 ± 1.2	285.30 ± 2.0	574.50 ± 1.7

Means marked in rows with different letters differ at *p* < 0.05.

**Table 4:** MIC and MBC values.

	Raw Material	Aqueous Dry Extract	Ethanolic Dry Extract
Strain	MIC (MBC) (mg/mL)		
Gram-positive bacteria			
*S. epidermidis* ATCC 12228	1 (4)	1 (8)	2 (8)
*S. aureus* ATCC 25923	4 (4)	4 (16)	1 (4)
*S. aureus* A 529	8 (8)	8 (>64)	2 (32)
*B. cereus* 15	16 (32)	16 (32)	4 (32)
*B. cereus* ATCC 11778	32 (64)	16 (16)	4 (4)
*B. cereus* X-13	32 (64)	16 (64)	4 (32)
*B. subtilis* ATCC 6633	32 (64)	16 (32)	2 (16)
*L. monocytogenes* 17/11	32 (>64)	8 (16)	4 (8)
Gram-negative bacteria			
*K. pneumoniae* ATCC 13883	2 (2)	1 (2)	1 (1)
*Y. enterocolitica* O3 383/11	4 (4)	4 (32)	4 (32)
*P. mirabilis* ATCC 35659	8 (64)	8 (16)	4 (16)
*S. sonnei* s	8 (16)	8 (64)	4 (64)
*S.* ser. Enteritidis ATCC 13076	16 (16)	8 (16)	8 (16)
*S*. ser. Enteritidis 322/11	32 (64)	16 (64)	16 (64)
*S*. ser. Typhimurium 300/11	32 (64)	16 (>64)	16 (32)
*Ps. aeruginosa* ATCC 27853	32 (64)	16 (32)	8 (16)
*E. aerogenes* ATCC 13048	32 (>64)	32 (64)	32 (64)
*E. coli* ATCC 25922	64 (>64)	32 (32)	32 (32)
*E. coli* O26 152/11	64 (64)	32 (64)	32 (64)

**Table 5:** Percentage of antibacterial activity (A %).

MIC (mg/mL)	A (%)		
	Raw Material	Aqueous Dry Extract	Ethanolic Dry Extract
0.5	0	0	0
1	5	11	26
2	11	11	26
4	21	21	63
8	37	47	74
16	47	84	84
32	90	100	100
64	100	100	100

**Table 6:** Validation parameters of the HPLC-DAD analysis (*n* = 6).

Compound	Precision Intra-Day (CV %)	Precision Inter-Day (CV %)	Regression Equation	R^2^ (*n* = 6)	Linear Range (mg mL^−1^)	LOD (µg L^−1^)	LOQ (µg L^−1^)	Recovery (%)
Salidroside	1.17	1.84	*y* = 231.6*x* + 1946.7	0.9999	3.9–3920.0	10.83	36.10	105.1
Tyrosol	1.22	1.74	*y* = 604.3*x* − 13205.1	0.9997	3.8–3800.0	31.73	105.78	92.5
Rosarin	0.95	1.32	*y* = 618.3*x* − 10440.9	0.9998	2.0–1960.0	7.31	24.37	98.0
Rosavin	0.80	1.22	*y* = 406.2*x* − 4640.1	0.9999	3.9–3872.0	7.69	25.65	95.4
Rosin	0.75	1.15	*y* = 480.6*x* − 11104.3	0.9996	2.9–1980.0	17.57	58.57	96.2
*Trans*-cinnamic alcohol	0.97	1.21	*y* = 7740.3*x* − 28673.2	0.9994	11.7–880.3	1.26	4.21	97.4
